# Neuromodulation by repetitive paired-pulse TMS at late I-wave intervals in older adults

**DOI:** 10.1007/s00221-025-07060-5

**Published:** 2025-05-06

**Authors:** Wei-Yeh Liao, Brodie J. Hand, Giuseppe Rinaldi, John G. Semmler, George M. Opie

**Affiliations:** 1 Discipline of Physiology, School of Biomedicine, The University of Adelaide, Adelaide, Australia; 2 Discipline of Anatomy and Pathology, School of Biomedicine, The University of Adelaide, Adelaide, Australia

**Keywords:** Transcranial magnetic stimulation, Ageing, Plasticity, Primary motor cortex

## Abstract

The efficacy of indirect (I) wave periodicity repetitive transcranial magnetic stimulation (iTMS) on the excitability of primary motor cortex (M1) in young adults can be modified by changing the late I-wave interval. Given that late I-wave activity is altered in older adults, this could contribute to age-related changes in M1 plasticity. To assess this possibility, the present study investigated the effects of iTMS using three late I-wave intervals (4.0, 4.5, and 5.0 ms) on M1 excitability in 17 older adults (69.6 ± 5.7 years; 10 females), which was compared to findings obtained previously in 17 young adults (27.2 ± 6.4 years, 12 females). Changes in M1 excitability were assessed using motor evoked potentials (MEPs) recorded from the right first dorsal interosseus to index single-pulse MEP_1.0mV_ and paired-pulse short-interval intracortical facilitation (SICF). To increase sensitivity to different intracortical circuits, both measures were also recorded using posterior-anterior (PA) and anterior-posterior (AP) TMS currents. Within older adults, PA MEP_1.0mV_ and SICF were facilitated following iTMS (both *P* < 0.0001) and these were not different between iTMS ISIs (both *P* > 0.077). In contrast, AP MEP_1.0mV_ and SICF were potentiated by iTMS_4.0_ and iTMS_5.0_ (both *P* < 0.023). iTMS_5.0_ potentiation of AP circuits was also increased in older adults compared to young adults (both *P* < 0.004). These results suggest complex, timing-dependent effects of advancing age on the plasticity of the late I-wave circuits.

## Introduction

The human brain continuously reorganises its intrinsic structure, connections and function throughout the lifespan (Bagarinao et al. 2019; Dinse 2006). This ability, termed neuroplasticity, underpins the generation of learning and memory with new experiences (Sanes and Donoghue 2000), which is important in regulating motor behaviour in day-to-day life (Buonomano and Merzenich 1998). Neuroplasticity has been extensively studied using transcranial magnetic stimulation (TMS), a type of non-invasive brain stimulation (NIBS) technique that is able to measure and induce short-term neuroplastic changes, particularly over primary motor cortex (M1) (Hallett 2000; Ziemann et al. 2008). While early TMS studies have demonstrated reduced M1 plasticity in older adults (Fathi et al. 2010; Freitas et al. 2011; Müller-Dahlhaus et al. 2008; Todd et al. 2010), more recent work has suggested that this is not always the case, with age-related TMS findings often being variable and difficult to interpret (Semmler et al. 2021). Importantly, the mechanisms driving this variability in M1 plasticity within older adults remain unclear.

TMS is a useful technique for probing different elements within the neuromotor system with high temporal resolution. Application of TMS over M1 generates a complex wave of descending volleys that summates at the spinal cord, which results in a motor evoked potential (MEP) in targeted muscles (Di Lazzaro et al. 1998). The first wave in this descending volley is thought to reflect direct (D-wave) activation of corticospinal neurons, whereas subsequent waves are thought to stem from indirect (I-wave) activation of local interneuronal networks synaptically connected to corticospinal neurons (Di Lazzaro et al. 1998). These I-waves occur with a periodicity of ~ 1.5 ms and can be further characterised as early (I1) and late (I2, I3), based on the order of appearance (Ziemann 2020). Early and late I-waves can be selectively recruited by changing the direction of the applied TMS current. For example, a posterior-anterior (PA) current preferentially recruits early I-waves, whereas an anterior-posterior (AP) current preferentially recruits late I-waves (Di Lazzaro et al. 2001; Hamada et al. 2013). Furthermore, the activity of I-wave circuits can be indexed using the paired-pulse TMS paradigm short-interval intracortical facilitation (SICF) (Tokimura et al. 1996; Ziemann et al. 1998). SICF produces a facilitated MEP following application of two TMS stimuli that are separated by interstimulus intervals (ISI) that approximate I-wave periodicity, allowing selective activation of early (~ 1.5 ms) or late (~ 4.5 ms) I-wave circuits (Ziemann et al. 1998). This facilitation is thought to stem from the second stimulus (S2) discharging I-wave-generating neurons that experienced subthreshold depolarisation following application of the first stimulus (S1) (Hanajima et al. 2002).

It is also possible to modulate the excitability of I-wave circuits by using the plasticity-inducing paradigm I-wave periodicity repetitive TMS (iTMS). This involves a low frequency (i.e., 0.2 Hz) train of 180 paired-pulse stimuli applied with ISIs approximating I-wave periodicity (Kidgell et al. 2016), with modification to ISI allowing the early (~ 1.5 ms) (Cash et al. 2009; Thickbroom et al. 2006) or late (~ 4.5 ms) (Hand et al. 2023; Long et al. 2017; Opie et al. 2018) I-waves to be targeted. Using iTMS, previous work has reported robust potentiation of MEP amplitude and SICF, which are thought to represent the induction of long-term potentiation-like effects (LTP) that mediate learning (Cash et al. 2009, 2011; Hand et al. 2023; Thickbroom et al. 2006). Importantly, recent work has demonstrated that applying iTMS can modulate interhemispheric interactions (Tian and Izumi 2023) and enhance visuomotor skill acquisition (Hand et al. 2023), highlighting the potential for the intervention to be implemented clinically. Together, these TMS techniques have established that the late I-wave circuits, in particular, play an important role in M1 plasticity and skilled motor learning (Hamada et al. 2013; Ho et al. 2022; Opie et al. 2021; Spampinato et al. 2020; Volz et al. 2019; Wiethoff et al. 2014).

Additionally, we have shown that the excitability and temporal characteristics of the late I-waves (I3) are related to the efficacy of iTMS in young adults (Opie et al. 2021). In particular, this study demonstrated that modifying the ISI approximating the late I-wave (I3) peak influences the efficacy of iTMS in M1 of young adults (Opie et al. 2021), suggesting that the timing of the late I-wave peak is important for the response to neuroplastic interventions. However, the characteristics of the late I-waves are altered in older adults (Opie et al. 2018, 2020), which may contribute to age-related changes in plasticity and motor learning. For example, we have previously shown that there is reduced excitability and delayed temporal characteristics of the late (I3) SICF peak in older adults. Furthermore, this age-related delay was correlated with changes in plasticity and aspects of motor behaviour in the elderly (Opie et al. 2018, 2020). Taken together, these findings suggest that age-related changes in the timing of late I-waves may contribute to the variable effects of age on M1 plasticity, but this has not been tested experimentally.

Therefore, the aim of the present study was to investigate whether age-related changes in the timing of late I-waves contribute to an altered neuroplastic response in older adults. This was achieved by assessing the response to iTMS applied with ISIs that encompass the typical late I-wave peak (4.0, 4.5, and 5.0 ms) via PA and AP measures of single-pulse MEPs and SICF in older adults, and comparing the data to previous findings in young adults (Opie et al. 2021). We hypothesised that the response to iTMS in older adults could be enhanced by selecting longer ISIs that match the temporally delayed late I-wave peaks.

## Materials and methods

### Participant sample

Sample size calculations were performed using simulation-based power estimations on Rstudio (version 2023.12.1 + 402) (Post-team) with ‘*lme4*’ (Bates 2010) and ‘*simr*’ (Green and MacLeod 2016) packages, and were based on data obtained previously in young adults using the same experimental procedure (Opie et al. 2021). Our a priori analysis revealed a fixed effects coefficient of 0.310 that was used as an unstandardised effect size of iTMS in young adults (Green and MacLeod 2016). This value was lowered by 15% (0.261) to account for biases in power calculations derived from experimental data (Kumle et al. 2021) and a potential weaker intervention effect in older adults. Given α < 0.05, β > 0.85 (0.894), a sample size of 13 participants was required to observe a small effect of iTMS. As AP responses were unattainable in 4 individuals, a total of 17 older adults were recruited (mean age ± standard deviation, 69.6 years ± 5.7 years; range, 62–79 years; 10 females). Participants were recruited via advertisements within The University of Adelaide, the wider community, and on social media platforms. Exclusion criteria included a history of neurological or psychiatric disease, left-handedness, or current use of medication that affect the central nervous system. Participant suitability for TMS was assessed via a standard TMS safety screening questionnaire (Rossi et al. 2011). The study was approved by the University of Adelaide Human Research Ethics Committee (H-026-2008) and conducted in accordance with the declaration of Helsinki. Each participant provided written, informed consent prior to participation.

### Experimental arrangement

**Fig. 1:**
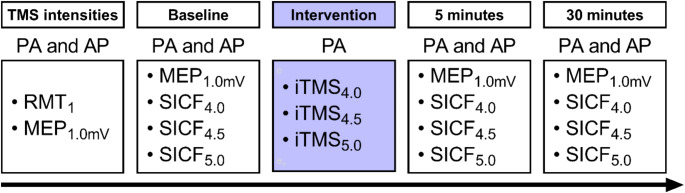
Experimental protocol. AP, anterior-posterior; iTMS, I-wave periodicity transcranial magnetic stimulation; MEP, motor evoked potential; PA, posterior-anterior; RMT, resting motor threshold; SICF, short-interval intracortical facilitation; TMS, transcranial magnetic stimulation

Participants attended three experimental sessions in a single-blind study design (Fig. 1), with a washout period between sessions of at least 7 days. During each session, participants were seated in a comfortable chair with their right arm and hand supported. Surface electromyography (EMG) was recorded from the first dorsal interosseous (FDI) muscle of the right hand using two Ag-AgCl electrodes arranged in a belly-tendon montage. A third electrode was placed over the styloid process to ground the electrodes. EMG signals were amplified (300x) and band-pass filtered (20 Hz high pass, 1 kHz low pass) using a CED1902 signal conditioner (Cambridge Electronic Design, Cambridge, UK) before being digitised at 2 kHz using a CED1401 analogue-to-digital converter (Cambridge Electronic Design, Cambridge, UK). Signal noise associated with mains power within the 50 Hz frequency band were removed via a Humbug mains noise eliminator (Quest Scientific, North Vancouver, Canada). Real-time EMG was displayed on an oscilloscope in front of the participant to facilitate muscle relaxation during each experimental session. Participant blinding was not assessed.

### Experimental procedures

#### Transcranial magnetic stimulation (TMS)

TMS was applied to left M1 using a branding iron coil (70 mm diameter) connected to two Magstim 200^2^ magnetic stimulators (Magstim, Whiteland, UK) via a BiStim unit. The coil was held tangentially to the scalp at an angle of 45° to the sagittal plane, inducing a PA current relative to the central sulcus. The M1 hotspot was identified as the location producing the largest and most consistent MEPs within the relaxed FDI. This location was marked on the scalp for reference and continuously monitored throughout each experimental session. All baseline and post-intervention (5 min, 30 min) TMS was applied at a rate of 0.2 Hz, with a 10% jitter between trials to avoid anticipation of the stimulus.

Resting motor threshold (RMT) over M1 was recorded each session as the lowest stimulus intensity (expressed as a percentage of maximum stimulator output; % MSO) required to produce an MEP amplitude ≥ 50 µV in 5 of 10 consecutive trials. Following RMT, the stimulus intensity required to produce an MEP amplitude ~ 0.5-1.0mV (MEP_1.0mV_), when averaged over 20 trials, was identified. These measures were then repeated using an AP current by rotating the coil 180° over the M1 hotspot. The same intensities for PA and AP MEP_1.0mV_ were used post-intervention.

#### Short-interval intracortical facilitation (SICF)

The paired-pulse measure SICF consisted of an initial stimulus (S1) set at MEP_1.0mV_ intensity and a second stimulus (S2) set at 90% RMT (Ziemann et al. 1998). In order to encapsulate the range of intervals associated with the third facilitatory peak of SICF, the ISIs 4.0 ms, 4.5 ms, and 5.0 ms were used. Each SICF block included 60 trials, divided into four conditions with 15 repetitions each (MEP_1.0mV_, SICF_4.0_, SICF_4.5_, SICF_5.0_). SICF was recorded at baseline, and 5- and 30-minutes post-intervention using PA and AP current directions.

#### I-wave periodicity TMS (iTMS)

In accordance with previous studies, iTMS consisted of 180 pairs of stimuli applied with a PA current at 0.2 Hz, totalling an intervention time of 15 min (Cash et al. 2009; Liao et al. 2022; Opie et al. 2021). The stimulation intensities were the same as PA SICF (S1, PA MEP_1.0mV_; S2, 90% PA RMT). The ISIs targeting the late I-waves (4.0, 4.5, and 5.0 ms) were applied in separate sessions, with the session order randomised between participants. To mitigate coil overheating, ice packs were applied over the coil prior to and during iTMS.

### Data analysis

All EMG data were recorded using CED Signal (v 6.02, Cambridge Electronic Design) and were visually inspected off-line, with any trials having EMG activity exceeding 30µV in the 100 ms prior to stimulus application excluded from analysis (approximately 8.31%). The amplitude of MEPs was measured peak-to-peak and expressed in mV. The individual paired-pulse MEP amplitude within baseline SICF was expressed as a percentage of the mean single-pulse test MEP amplitude within the same block. For all post-iTMS SICF, individual paired-pulse MEP amplitude was expressed as a percentage of the mean single-pulse MEP amplitude recorded at baseline, as undertaken previously (Cash et al. 2009; Liao et al. 2022, 2023; Opie et al. 2021). This was performed as the increase in post-iTMS single-pulse MEP amplitude is correlated with the increase in post-intervention SICF MEP amplitude, and normalising to the mean post-intervention test MEP amplitude underestimates the changes in excitability of the I-wave circuits (Cash et al. 2009). This relationship was also tested in the present study, which revealed a significant correlation between the increase in post-iTMS single-pulse MEP amplitude and SICF MEP amplitude (ρ = 0.75, *P* = 0.001). MEP amplitude recorded during each iTMS block were split into 10 blocks with 18 consecutive MEP trials each.

Furthermore, baseline single-pulse MEP_1.0mV_ latency (obtained in the resting muscle) was assessed for young and older adults using a semi-automated process via a custom script within CED Signal. MEP_1.0mV_ latency within each trial was defined as the point at which the rectified EMG signal following the stimulus artefact exceeded the mean EMG amplitude plus 2 SD within the 100 ms pre-stimulus period. Trials with stimulus artefacts that contaminated the EMG signal were excluded from analysis (approximately 22.0%). PA and AP MEP_1.0mV_ latency was averaged for each participant within each session and expressed in ms.

### Statistical analysis

Statistical analyses were performed using the ‘*lme4*’ package on Rstudio. All MEP data were compared between conditions using generalised linear mixed models (GLMMs) with gamma distributions and log link functions, which are suitable for positively-skewed MEP data (Liao et al. 2022, 2023; Lo and Andrews 2015; Puri and Hinder 2022). Each model included random participant intercepts and model fit was assessed by the Bayesian Schwartz criterion (BIC). Our statistical analysis was split into two parts: a primary analysis investigating the effects of iTMS in older adults alone, and a secondary analysis characterising how these responses differ from young adults. For the primary analysis, MEP amplitude during the iTMS block was investigated using a two-factor model assessing the effects of iTMS session (iTMS_4.0_, iTMS_4.5_, iTMS_5.0_) and iTMS block (iTMS blocks; B1-B10). The effects of iTMS on PA and AP MEP_1.0mV_ amplitude during each session were investigated using two two-factor models assessing the effects of iTMS session and time point (baseline, 5 min, 30 min). The effects of iTMS on PA and AP SICF during each session were investigated using two three-factor models assessing the effects of iTMS session, time point, and SICF ISI (4.0ms, 4.5ms, 5.0ms).

Our secondary analysis investigating age-related differences in the effects of iTMS was achieved by comparing data from older adults of the current study with a previous dataset of young adults from our group (Opie et al. 2021). Models for the secondary analysis were analogous to those applied within the primary analysis, except for the addition of an age group factor (young, older). Furthermore, a three-factor model was used to investigate the effects of iTMS session, coil orientation (PA, AP), and age group on mean baseline PA and AP MEP_1.0mV_ latency. For each model investigating data of both young and older adults, the random participant intercepts were nested within studies (young iTMS, older iTMS). To control for between-group differences in baseline variability, and retain consistency with published results, data from the secondary analysis are expressed as a ratio of the baseline response (see below for details on how this was derived).

**Fig. 2:**
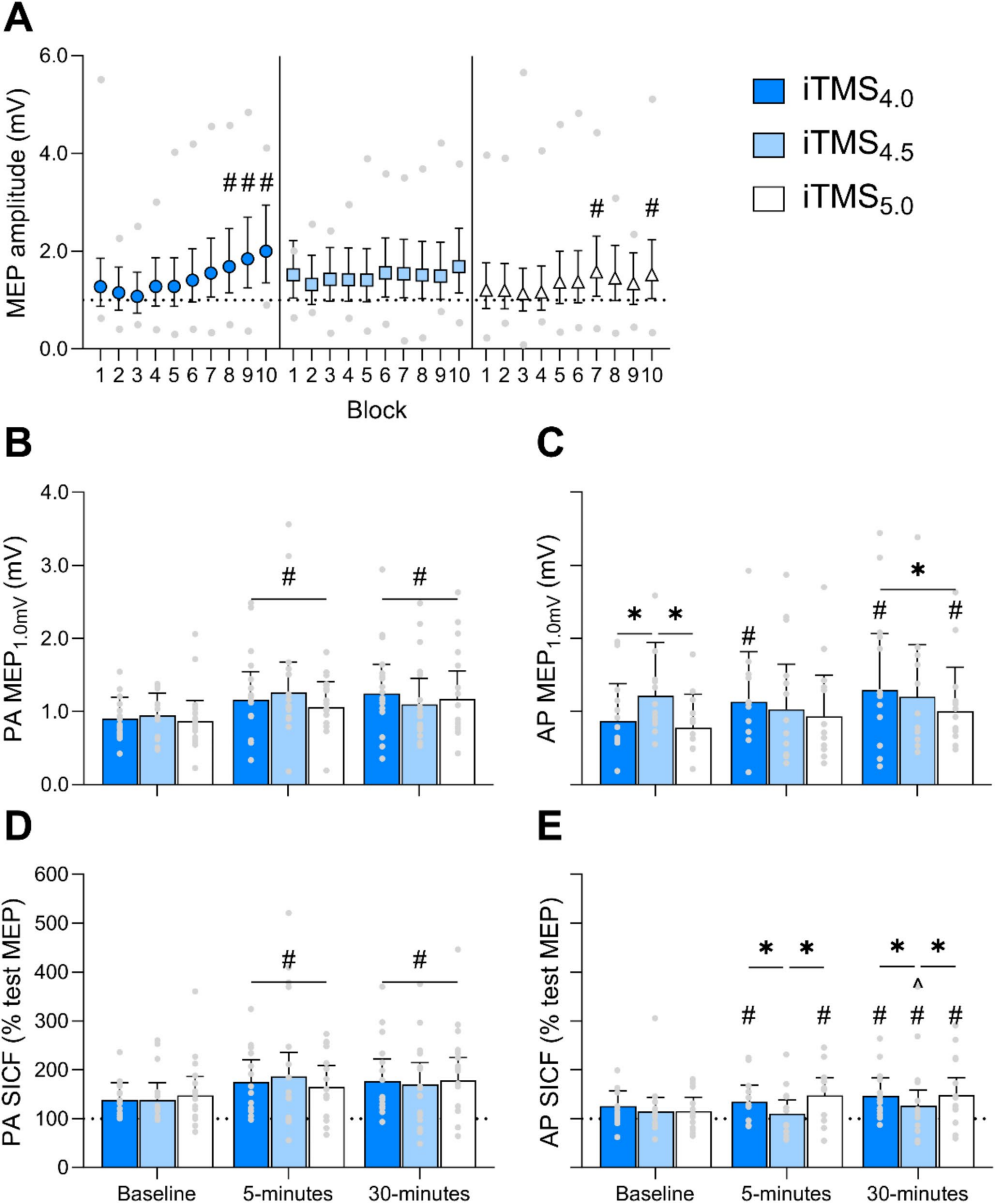
On-line and off-line effects of iTMS_4.0_ (blue), iTMS_4.5_ (light blue) and iTMS_5.0_ (white) in older adults. (**A**) MEP amplitude during iTMS blocks 1–10. (**B**) PA MEP_1.0mV_, (**C**) AP MEP_1.0mV_, (**D**) PA SICF and (**E**) AP SICF at baseline, 5 and 30 min post-iTMS. Data are presented as EMM [95% CI] with (**A**) minimum and maximum participant means, and (B-E) individual participant means. *P < 0.05. #*P* < 0.05 compared to baseline/B1. ^*P* < 0.05 compared to 5-minutes

**Fig. 3:**
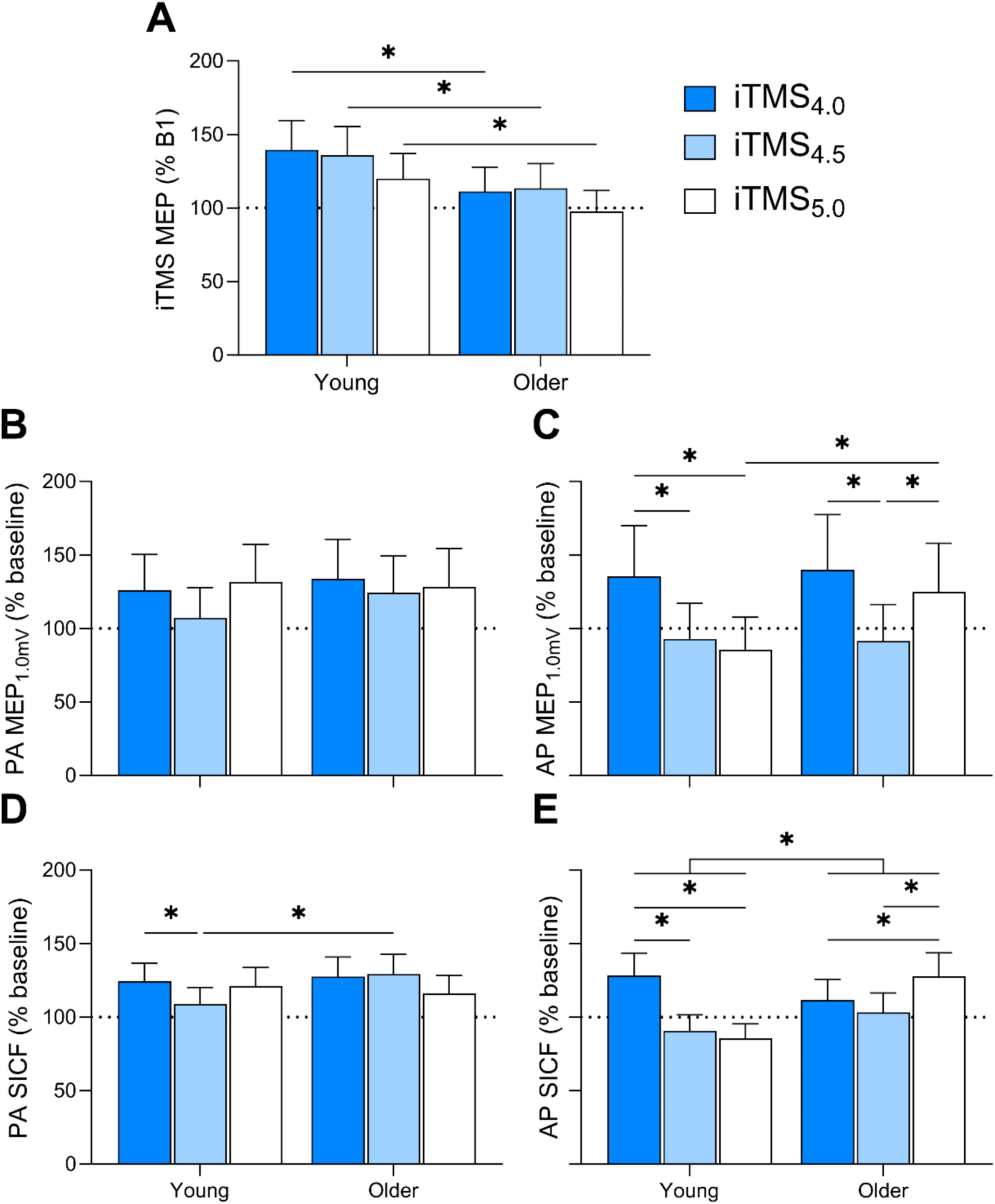
Changes in on-line and off-line effects of iTMS_4.0_ (blue), iTMS_4.5_ (light blue) and iTMS_5.0_ (white) in young and older adults. (**A**) MEP amplitude during iTMS blocks 1–10. (**B**) PA MEP_1.0mV_, (**C**) AP MEP_1.0mV_, (**D**) PA SICF and (**E**) AP SICF at baseline, 5 and 30 min post-iTMS. Data are presented as EMM [95% CI]. *P < 0.05

Following model fitting, we planned custom *post-hoc* contrasts using the ‘*emmeans*’ package (Lenth 2022) to probe significant main effects and interactions. We first investigated the effects of the intervention within older adults (see Results, Fig. 2). We then compared the effects of the intervention between young and older adults (see Results, Fig. 3). For on-line age-related effects, this was achieved by expressing the mean MEP amplitude from B2-10 as a ratio of B1 within the model, and comparing these ratios between sessions and age groups. For off-line age-related effects, the mean post-intervention PA and AP measures of MEP_1.0mV_ amplitude and SICF were expressed as a ratio of baseline responses within each model before comparing between sessions and age groups. Finally, we tested whether baseline MEP_1.0mV_ latency and SICF at each ISI varied between young and older adults. All *post-hoc* analyses were adjusted with Bonferroni corrections to control for type-I errors. All models are presented as estimated marginal means (EMM) with 95% confidence intervals (95% CI), and pairwise comparisons are presented as estimated mean differences (EMD) with 95% CI for unstandardised measures of effect size.

As undertaken previously in young adults (Opie et al. 2021), Spearman’s rank order correlation analysis was used to assess the relationship between the magnitude of SICF recorded within each ISI at baseline and the response to iTMS measured within the same session for older adults. In addition, the correlation between maximum SICF at baseline (identified numerically with visual inspection of the data) and the response to iTMS within the same session was assessed in older adults. Correlations are presented as Spearman’s ρ, and significance was adjusted using Bonferroni corrections. For all models, *P* < 0.05 was considered significant.

## Results

All participants completed the three experimental sessions without adverse effects. We were unable to record AP MEPs in four older participants (3 female) due to high threshold of activation (AP RMT not recordable). In addition, we were unable to record PA MEPs in one older male participant 5 min following iTMS due to coil overheating. To more clearly address the specific interests of the current study, the results will first detail effects within the older group. Age-related comparisons relative to our previously published dataset (Opie et al. 2021) will then be reported.

### Effects of iTMS in older adults

#### Baseline

Baseline stimulation intensities, MEP amplitude and SICF are presented in Table 1. There were no differences between sessions for all stimulation intensities (all *P* > 0.355). Baseline AP MEP_1.0mV_ was increased during iTMS_4.5_ session compared to iTMS_4.0_ (EMD = 40.2% [0.4, 98.7], *P* = 0.0007) and iTMS_5.0_ (EMD = 56.5% [12.4, 118.1], *P* < 0.0001). MEP amplitude during B1 of iTMS_4.5_ was also increased compared to iTMS_4.0_ (EMD = 11.6% [-5.6, 31.8], *P* = 0.014) and iTMS_5.0_ (EMD = 26.0% [-3.0, 63.7], *P* = 0.0006). There were no other significant effects at baseline (all *P* > 0.416).

**Table 1:** Baseline TMS intensities, MEP1.0mV, SICF, and first block of iTMS between sessions of older adults

Measure		iTMS_4.0_	iTMS_4.5_	iTMS_5.0_
PA				
PA RMT (%MSO)		47.5 [45.5, 49.5]	46.5 [44.6, 48.5]	47.8 [45.8, 49.8]
PA MEP_1.0mV_ (%MSO)		62.6 [59.1, 66.2]	62.8 [59.4, 66.4]	63.3 [59.8, 66.9]
PA MEP_1.0mV_ (mV)		0.90 [0.76, 1.07]	0.95 [0.80, 1.12]	0.87 [0.74, 1.03]
PA SICF (% Test)				
	4.0 ms ISI	123.6 [88.7, 172.4]	117.2 [84.1, 163.5]	130.7 [93.8, 182.1]
	4.5 ms ISI	150.0 [128.3, 175.4]	151.8 [129.7, 177.6]	157.1 [134.3, 183.8]
	5.0 ms ISI	141.3 [120.8, 165.5]	147.3 [125.9, 172.4]	156.6 [133.8, 183.3]
**AP**				
AP RMT (% MSO)		61.5 [58.5, 64.4]	60.7 [57.7, 63.6]	61.6 [58.7, 64.6]
AP MEP_1.0mV_ (% MSO)		78.4 [74.0, 82.8]	78.3 [73.9, 82.7]	78.6 [74.2, 83.0]
AP MEP_1.0mV_ (mV)		0.87 [0.67, 1.12]^a^	1.22 [0.94, 1.58]	0.78 [0.60, 1.01]^a^
AP SICF (% Test)				
	4.0 ms ISI	116.9 [100.8, 135.6]	117.0 [100.6, 136.0]	102.8 [88.5, 119.4]
	4.5 ms ISI	131.0 [112.8, 152.1]	117.7 [101.3, 136.8]	134.0 [115.7, 155.2]
	5.0 ms ISI	130.0 [111.9, 150.9]	112.4 [97.2, 129.9]	109.7 [94.3, 127.5]
**iTMS**				
iTMS block 1 (mV)		1.27 [0.86, 1.88]	1.52 [1.03, 2.25]	1.20 [0.81, 1.78]^a^
Data are presented as EMM [95% CI; lower, upper].				

#### Corticospinal excitability during iTMS

MEP amplitude during iTMS differed between sessions (X^2^ (2) = 14.5, *P* = 0.0007), with increased MEP amplitude during iTMS_4.0_ (EMD = 7.6% [1.9, 13.7], *P* = 0.001) and iTMS_4.5_ (EMD = 12.0% [6.1, 18.2], *P* < 0.0001) compared to iTMS_5.0_. MEP amplitude during iTMS also varied between blocks (X^2^ (9) = 170.1, *P* < 0.0001), with increased MEP amplitude during blocks 6–10 compared to block 1 (all *P* < 0.002). There were also a two-factor interaction between session and block (X^2^ (18) = 71.5, *P* < 0.0001; Fig. 2A). *Post-hoc* analysis for the interaction revealed increased MEP amplitude during B8-10 of iTMS_4.0_ compared to B1 (all *P* < 0.0006) and during B7 and B10 of iTMS_5.0_ compared to B1 (both *P* < 0. 021).

#### Corticospinal excitability following iTMS

PA MEP_1.0mV_ amplitude did not vary between sessions (X^2^ (2) = 1.5, *P* = 0.473), but varied between time points (X^2^ (2) = 23.1, *P* < 0.0001), with increased MEP amplitude relative to baseline following the intervention (both time points *P* < 0.0001). There was no interaction between session and time point (X^2^ (4) = 7.4, *P* = 0.115; Fig. 2B).

AP MEP_1.0mV_ amplitude differed between sessions (X^2^ (2) = 27.0, *P* < 0.0001), with increased MEP amplitude during iTMS_4.0_ (EMD = 20.4% [4.9, 38.2], *P* = 0.001) and iTMS_4.5_ (EMD = 27.2% [10.8, 46.0], *P* < 0.0001) compared to iTMS_5.0_. AP MEP_1.0mV_ amplitude also varied between time points (X^2^ (2) = 21.3, *P* < 0.0001), with increased MEP amplitude 30 min following iTMS compared to baseline (EMD = 24.1% [8.3, 42.2], *P* = 0.0001). There was also a session by time point interaction (X^2^ (4) = 16.3, *P* = 0.003; Fig. 2C), which revealed increased AP MEP_1.0mV_ amplitude relative to baseline at both time points following iTMS_4.0_ (both *P* < 0.01) and 30 min following iTMS_5.0_ (EMD = 29.7% [-2.6, 72.6], *P* = 0.009). AP MEP_1.0mV_ amplitude 30 min following iTMS_4.0_ was also increased compared to iTMS_5.0_ (EMD = 28.7% [-3.5, 71.5], *P* = 0.013).

#### Intracortical excitability following iTMS in older adults

PA SICF did not vary between sessions (X^2^ (2) = 2.7, *P* = 0.258), but differed between time points (X^2^ (2) = 20.6, *P* < 0.0001), with increased SICF at both post-iTMS time points compared to baseline (both *P* < 0.0001). PA SICF varied between SICF ISIs (X^2^ (2) = 9.0, *P* = 0.011), with increased PA SICF at 4.5 ms (EMD = 16.3% [9.6, 23.4], *P* < 0.0001) and 5.0 ms (EMD = 11.9% [5.5, 18.7], *P* < 0.0001) compared to 4.0 ms. There were no interactions between factors (all *P* > 0.062; Fig. 2D shows session by time point values).

AP SICF did not differ between sessions (X^2^ (2) = 1.2, *P* = 0.555) or SICF ISI (X^2^ (2) = 1.27, *P* = 0.529), but varied between time points (X^2^ (2) = 10.5, *P* = 0.005), with responses increasing throughout each session (all *P* < 0.033). There was also a session by time point effect (X^2^ (4) = 15.5, *P* = 0.004; Fig. 2E), with *post-hoc* contrasts showing increased AP SICF at both time points following iTMS_4.0_ and iTMS_5.0_ compared to baseline (all *P* < 0.008), and increased AP SICF 30 min following iTMS_4.5_ compared to 5-minutes (EMD = 16.5% [-1.4, 37.8], *P* = 0.009) and baseline (EMD = 13.4% [-4.0, 34.0], *P* = 0.041). AP SICF was also increased following iTMS_4.0_ and iTMS_5.0_ relative to iTMS_4.5_ at both time points (all *P* < 0.036). There were no other interactions between factors (all *P* > 0.107).

### Age-related differences in the effects of iTMS

#### Age-related differences in corticospinal excitability during iTMS

MEP amplitude during iTMS did not vary between age groups (X^2^ (1) = 3.0, *P* = 0.081), but differed between sessions (X^2^ (2) = 15.1, *P* = 0.0005) and between blocks (X^2^ (9) = 176.3, *P* < 0.0001). There were also two-factor interactions between session and block (X^2^ (18) = 74.1, *P* < 0.0001), age and block (X^2^ (9) = 25.5, *P* = 0.002), and a three-factor interaction (X^2^ (18) = 39.2, *P* = 0.003). There was no interaction between session and age group (X^2^ (2) = 4.6, *P* = 0.1). For all iTMS sessions, on-line changes in the mean MEP amplitude during blocks 2–10 were reduced in older adults compared to young adults (all *P* < 0.005; Fig. 3A).

#### Age-related differences in corticospinal excitability following iTMS

PA MEP_1.0mV_ amplitude (Fig. 3B) did not vary between sessions (X^2^ (2) = 5.6, *P* = 0.062) or age groups (X^2^ (1) = 2.2, *P* = 0.141), but varied between time points (X^2^ (2) = 79.0, *P* < 0.0001). In addition, there was an interaction between session and time point (X^2^ (4) = 12.1, *P* = 0.017). Given these effects failed to interact with the age factor, post-hoc comparisons will not be described in detail. There were no other interactions between factors (*P* > 0.1).

AP MEP_1.0mV_ amplitude did not vary between age groups (X^2^ (1) = 3.7, *P* = 0.053), but differed between sessions (X^2^ (2) = 44.8, *P* < 0.0001) and between time points (X^2^ (2) = 15.6, *P* = 0.0004). There was also a session by age group interaction (X^2^ (2) = 21.1, *P* < 0.0001), a session by time point interaction (X^2^ (4) = 30.1, *P* < 0.0001), and a three-factor interaction between session, time point, and age group (X^2^ (4) = 11.4, *P* = 0.023). There was no interaction between time and age group (*P* > 0.09). Post-hoc comparisons for the three-factor interaction showed that off-line changes in AP MEP_1.0mV_ amplitude (Fig. 3C) were increased following iTMS_4.0_ compared to iTMS_4.5_ and iTMS_5.0_ in young adults (both *P* < 0.002), as reported previously (Opie et al. 2021). In contrast, off-line changes in AP MEP_1.0mV_ amplitude of older adults were increased following both iTMS_4.0_ (EMD = 36.6% [-2.1, 91.3], *P* = 0.017) and iTMS_5.0_ (EMD = 53.1% [9.2, 114.6], *P* = 0.002) compared to iTMS_4.5_. Off-line changes AP MEP_1.0mV_ amplitude following iTMS_5.0_ were also increased in older adults compared to young (EMD = 45.8% [5.6, 101.1], *P* = 0.0006).

#### Age-related differences in intracortical excitability following iTMS

SICF ISIs at baseline did not vary between young and older adults (all *P* > 0.055). PA SICF differed between sessions (X^2^ (2) = 9.2, *P* = 0.010), time points (X^2^ (2) = 194.9, *P* < 0.0001), SICF ISIs (X^2^ (2) = 44.6, *P* < 0.0001), but not between age groups (X^2^ (1) = 3.8, *P* = 0.052). There was a session by age group interaction (X^2^ (2) = 6.7, *P* = 0.034), an age group by SICF ISI interaction (X^2^ (2) = 18.1, *P* = 0.0001), and a three-factor interaction between session, time point, and age group (X^2^ (4) = 20.8, *P* = 0.0003). There were no other interactions between factors (*P* > 0.06). Post-hoc comparisons for the three-factor interaction showed that off-line changes in PA SICF (Fig. 3D) of young adults following iTMS_4.0_ were increased compared to iTMS_4.5_ (EMD = 13.9% [-0.8, 30.7], *P* = 0.014). In contrast, off-line changes in PA SICF of older adults following iTMS_4.5_ were increased compared to young adults (EMD = 18.5% [3.2, 35.9], *P* = 0.0003).

AP SICF varied between sessions (X^2^ (2) = 48.1, *P* < 0.0001), time points (X^2^ (2) = 16.8, *P* = 0.0002), SICF ISIs (X^2^ (2) = 23.8, *P* < 0.0001), but not between age groups (X^2^ (1) = 0.3, *P* = 0.600). There was a session by age group effect (X^2^ (2) = 64.1, *P* < 0.0001), a session by time point effect (X^2^ (4) = 43.0, *P* < 0.0001), an age group by time point effect (X^2^ (2) = 23.2, *P* < 0.0001), a time point by SICF ISI effect (X^2^ (4) = 15.7, *P* = 0.003), and a three-factor interaction between session, age group, and time point (X^2^ (4) = 73.4, *P* < 0.0001). There were no interactions between other factors (all *P* > 0.051). Post-hoc comparisons for the three-factor interaction showed that off-line changes in AP SICF (Fig. 3E) of young adults following iTMS_4.0_ were increased compared to iTMS_4.5_ (EMD = 41.4% [20.6, 65.7], *P* < 0.0001) and iTMS_5.0_ (EMD = 49.9% [28.1, 75.5], *P* < 0.0001). In contrast, AP SICF in older adults was increased following iTMS_5.0_ compared to iTMS_4.0_ (EMD = 14.6% [3.2, 35.9], *P* = 0.04) and iTMS_4.5_ (EMD = 24.1% [4.9, 28.4], *P* = 0.0004). Importantly, while off-line changes in AP SICF of young adults were increased compared to older adults following iTMS_4.0_ (EMD = 15.0% [-2.1, 35.1], *P* = 0.011), AP SICF was increased in older adults compared to young following iTMS_4.5_ (EMD = 13.5% [-3.5, 33.5], *P* = 0.022) and iTMS_5.0_ (EMD = 49.4% [27.7, 74.9], *P* < 0.0001).

#### Age-related differences in MEP onset latency at baseline

Baseline MEP latencies (Table 2) varied between age groups (X^2^ (1) = 23.1, *P* < 0.0001) and coil orientations (X^2^ (1) = 54.0, *P* < 0.0001), with comparisons revealing longer MEP latencies in older adults compared to young (EMD = 2.4 ms [1.1, 3.6], *P* = 0.0001), and longer AP MEP latencies compared to PA (EMD = 1.9 ms [1.2, 2.5], *P* < 0.0001).

**Table 2:** Baseline MEP latencies between sessions of young and older adults

Measure	iTMS_4.0_	iTMS_4.5_	iTMS_5.0_
Young			
PA MEP_1.0mV_ (ms)	21.1 [20.3, 21.9]	21.1 [20.4, 21.9]	21.2 [20.4, 22.0]
AP MEP_1.0mV_ (ms)^a^	23.1 [22.3, 23.9]	23.1 [22.3, 23.9]	23.2 [22.4, 24.0]
**Older** ^b^			
PA MEP_1.0mV_ (ms)	23.9 [23.1, 24.7]	23.5 [22.7, 24.3]	23.6 [22.8, 24.4]
AP MEP_1.0mV_ (ms)^a^	25.6 [24.6, 26.5]	25.4 [24.4, 26.3]	25.1 [24.2, 26.1]
Data are presented as EMM [95% CI; lower, upper]. ^a^*P* < 0.05 compared to PA. ^b^*P* < 0.05 compared to young.			

### Correlation analyses

Spearman’s rank correlation analysis did not reveal a relationship between the magnitude of baseline SICF recorded within each ISI and changes in corticospinal or intracortical excitability following iTMS within the same session for older adults (all ρ < 0.676, *P* > 0.264). There was also no correlation between maximum baseline SICF and changes in corticospinal or intracortical excitability following iTMS of the same session (all ρ < 0.516, *P* > 0.071).

## Discussion

We have previously shown that the temporal characteristics of the late I-waves are altered in older adults (Opie et al. 2018, 2020), but the extent to which these changes influence neuroplasticity in the elderly has remained unclear. To address this limitation, the current study applied iTMS in older adults using three ISIs (4.0, 4.5, and 5.0 ms) that encapsulate the prototypical timing of the third I-wave peak, and examined changes in PA and AP measures of MEP_1.0mV_ and SICF. For both MEPs and SICF, this revealed potentiation of PA responses that were invariant to iTMS timing, whereas potentiation of AP responses differed between iTMS intervals. Furthermore, a secondary analysis comparing the effects in older adults to those seen previously in a young cohort highlighted age-related differences that were specific to AP responses. Taken together, these findings demonstrate that effects of age on the late I-wave circuits are complex, varying between the specific circuits tested.

### Effects of iTMS in older adults

Application of iTMS over M1 has been shown to produce robust on-line and off-line potentiation of M1 excitability in young adults (Cash et al. 2009; Thickbroom et al. 2006). This effect is thought to stem from the induction of LTP-like events involving the modulation of I-wave circuits (Thickbroom et al. 2006). Importantly, activation of these I-wave circuits is dependent on the ISIs used during paired-pulse TMS (Di Lazzaro et al. 1999; Wagle-Shukla et al. 2009), and varying these ISIs has been shown to influence the efficacy of iTMS targeting early or late I-waves in young adults (Opie et al. 2021; Sewerin et al. 2011). As this variance in the efficacy of the intervention seems to be related to the temporal characteristics of the targeted circuits (Opie et al. 2021; Sewerin et al. 2011), we reasoned that age-related delays in the temporal characteristics of the late I-wave circuits (Opie et al. 2018, 2020) may play an important role in changes to neuroplasticity within the elderly (Fathi et al. 2010; Freitas et al. 2011; Müller-Dahlhaus et al. 2008; Todd et al. 2010). Specifically, if reduced plasticity responses stemmed from poorly aligned stimulation timing, we would expect that iTMS with longer ISIs would result in a greater neuroplastic response (Opie et al. 2018). However, our primary analysis of responses in older adults did not provide evidence to support this hypothesis. Specifically, the facilitation of PA TMS measures in older adults were not different between iTMS intervals. Furthermore, although AP responses did vary between iTMS intervals, the effects were not strong, and the pattern of response was not consistent with the proposed mechanism: increased facilitation tended to be apparent at shorter iTMS intervals (MEPs following iTMS_4.0_; Fig. 2C), or both shorter and longer iTMS intervals (SICF following iTMS_4.0_ and iTMS_5.0_; Fig. 2E).

These results suggest that adjusting iTMS intervals does not influence neuroplasticity induction in older adults. However, it is important to note that our initial hypothesis relied on the assumption that temporal delays within the late I-waves are a consistent feature in older adults, which has been demonstrated by our group and others (Clark et al. 2011; Opie et al. 2018, 2020; Rurak et al. 2019). Therefore, the absence of any significant temporal delays for PA and AP stimulation within older adults represents a significant confound of the current study’s ability to test its hypothesis. It will be important to further investigate the factors that contribute to timing variability within the late I-waves of older adults. While the absence of this effect reflects a limitation of the current study, this type of variability could be viewed as a positive from the perspective of potential biomarkers. For example, further defining how variability in late I-wave timing relates to the functional roles of the late I-waves– reported by our group (Hand et al. 2023; Opie et al. 2020) and others (Hamada et al. 2014; Spampinato et al. 2020)– will help establish potential clinical utility.

### Age-related changes in the response to iTMS

While our primary analysis allowed us to characterise how iTMS timing might influence M1 plasticity response in the elderly, contextualising this effect within the broader literature that has examined age-related changes in plasticity required comparisons with a younger cohort. To achieve this, we utilised data from an earlier study in which younger adults underwent an identical protocol (Opie et al. 2021), to perform a secondary analysis that directly compared responses between young and older adults (see Fig. 3). In an attempt to address some limitations stemming from baseline differences (see below), these data were compared between groups as ratios of baseline values (i.e., baseline-normalised). Considering on-line responses recorded during iTMS, this analysis found significant age-related reductions in response amplitude that were invariant to iTMS timing. Previous literature has interpreted online potentiation of MEP amplitude as indicative of neuroplastic effects (Cash et al. 2009; Thickbroom et al. 2006), suggesting that this difference between groups reflects a generalised reduction in neuroplasticity in the current older cohort. However, interpretation of this outcome is complicated by post-intervention PA MEPs and SICF, which showed responses that were generally comparable between groups (although we note slightly greater potentiation of PA SICF in older adults following iTMS_4.5_). Given that changes persisting beyond cessation of stimulation are considered prototypical of a plasticity intervention (Hallett 2007), we believe that post-intervention PA responses indicate that older adults maintain a level of plasticity within PA circuits that is comparable to that of younger adults. Importantly though, both on-line and off-line responses agree that plasticity within PA-circuits was invariant to iTMS timing, supporting the findings of the primary analysis.

For AP MEPs, facilitation in older adults was apparent following both iTMS_4.0_ and iTMS_5.0_, with responses to iTMS_5.0_ being significantly increased compared to young adults. While this could suggest that age-related changes in the plasticity response may be dependent on temporal characteristics of the intervention, interpretation is complicated by two points. First, baseline AP MEP amplitude was significantly increased in the iTMS_4.5_ condition. Consequently, ceiling effects may have prevented facilitation in response to iTMS_4.5_ that, if present, would suggest as temporally non-specific response to iTMS (as opposed to the temporally specific response shown by the current data). Second, facilitation of AP SICF within older adults was only apparent following iTMS_5.0_. While this would also be consistent with a temporally specific change in plasticity of older adults, the differences in timing (i.e., iTMS_4.0_ and iTMS_5.0_ for AP MEPs vs. iTMS_5.0_ for AP SICF) are difficult to reconcile. One explanation could be that MEPs involve overlapping recruitment of early and late I-waves (Di Lazzaro et al. 2001) whereas SICF is likely more sensitive to specific I-wave circuits (Opie and Semmler 2021). Despite this, differences in AP SICF between iTMS conditions were relatively small (i.e., < 15% for iTMS_4.0_ vs. iTMS_5.0_), and the potential relevance of this effect will require substantiation in future work. Irrespective of these details around timing, the absolute magnitude of facilitation for both AP MEPs and SICF was comparable between groups, suggesting that plasticity of AP circuits was also maintained in this cohort of older adults.

In conclusion, the current study investigated how age-related changes in the late I-waves influence neuroplasticity in older adults. A primary analysis of how older adults responded to iTMS at different late I-wave intervals suggested PA responses were temporally invariant, whereas AP responses tended to show unexpected patterns of facilitation. These effects were further examined in a secondary analysis comparing data from older adults of the current study to responses from a previously published young cohort. This analysis further supported the temporally invariant nature of PA responses in the elderly, while additionally demonstrating that plasticity of PA circuits was maintained in the older group. Secondary analysis of AP responses identified effects of stimulus timing on the plasticity response in older adults, but further work controlling for some important limitations is needed to clarify this outcome. Taken together, results were inconsistent with hypotheses relating stimulus timing to plasticity of late I-wave circuits in older adults, or inconclusive in nature.

## Data Availability

Data from this study will be made available to qualified investigators upon reasonable request to the corresponding author.
